# Ecological Niche Models of Four Hard Tick Genera (Ixodidae) in Mexico

**DOI:** 10.3390/ani10040649

**Published:** 2020-04-09

**Authors:** Emilio Clarke-Crespo, Claudia N. Moreno-Arzate, Carlos A. López-González

**Affiliations:** 1 Tecnológico de Monterrey, Escuela de Ingeniería y Ciencias, Centro de Bioingeniería, Queretaro 76130, Mexico; 2 Facultad de Ciencias Naturales, Universidad Autónoma de Querétaro, Queretaro 76230, Mexico; 3 Instituto de Ecología, Universidad Nacional Autónoma de México, Mexico City 04510, Mexico

**Keywords:** *Amblyomma*, *Dermacentor*, *Ixodes*, *Rhipicephalus*, ecological niche modelling

## Abstract

**Simple Summary:**

Vector-borne diseases currently represent a significant threat to public health, mainly due to the changes that humans are producing in ecosystems and climates. Analyzing the environmental conditions that allow the establishment and survival of ticks could help determine possible sites for the appearance of infectious outbreaks. In this study, nine ecological niche models were generated from different algorithms to determine the current potential distribution of four tick genera in Mexico. Temperature and moisture have been considered as the main factors limiting tick distribution. However, the analysis of the ecological niche models determined that the four genera exhibited different distribution patterns, which may be associated with their physiological and ecological differences. This type of analysis can improve our understanding of the dynamics of ticks and, therefore, can be very useful in monitoring programs of the diseases they transmit.

**Abstract:**

Ticks are vectors of a large number of pathogens of medical and veterinary importance, and in recent years, they have participated in the rise of multiple infectious outbreaks around the world. Studies have proposed that temperature and precipitation are the main variables that limit the geographical distribution of ticks. The analysis of environmental constraints with ecological niche modeling (ENM) techniques can improve our ability to identify suitable areas for emergence events. Algorithms used in this study showed different distributional patterns for each tick genera; the environmental suitability for *Amblyomma* includes warm and humid localities below 1000 m above the sea level, while *Ixodes* is mainly associated with ecosystems with high vegetation cover. *Dermacentor* and *Rhipicephalus* genus presented wider distribution patterns; the first includes species that are well adapted to resist desiccation, whereas the latter includes generalist species that are mostly associated with domestic hosts in Mexico. Ecological niche models have proven to be useful in estimating the geographic distribution of many taxa of ticks. Despite our limited knowledge of tick’s diversity, ENM can improve our understanding of the dynamics of vector-borne diseases and can assist public health decision-making processes.

## 1. Introduction

Vector-borne diseases are complex systems that require the interactions between arthropod vectors hosts and pathogens, constrained by a set of environmental variables [1]. Particularly, vector reproduction, dispersal, and survival are strongly influenced by environmental conditions [2]. Ecological niche models (ENMs) have become powerful tools in the study of vector ecology. These are mainly used to determine their current and/or future potential distribution, to identify risk areas prone to the emergence of infectious outbreaks, or to distinguish environmental variables that regulate the dynamics of infectious diseases at a landscape level [3]. When applied to the estimation of potential geographic distribution, ecological niche models identify nonrandom patterns that usually emerge from the superposition analysis of a collection of presence-only or presence/absence data with a set of environmental variables [4,5] using different machine learning techniques [6,7].

An important arthropod disease carrier is ticks, which, in addition to being hematophagous ectoparasites of wild and domesticated animals and humans, are also known to be key vectors of a great variety of pathogens, such as protozoa, rickettsia, spirochaetes, and viruses [8,9,10]. Human-induced environmental changes appear to be important drivers that enhance tick distribution and survival. These vectors have triggered important human outbreaks such as tick-borne encephalitis (TBE) in Europe, Kyasanur forest disease (KFD) in India, Crimean-Congo hemorrhagic fever (CCHF) in Turkey and Russia, Q fever in the Netherlands, and Rocky Mountain spotted fever (RMSF) in the southern United States and in northern Mexico [11,12,13]. Overall, these events have supported the recognition of tick and tick-borne diseases as important emerging threats to humans and animals [14,15,16].

Hard ticks’ survival off hosts relies heavily on their ability to resist desiccation either by physiological or behavioral adaptations or by the environmental characteristics of the ecosystem in which they are found. For example, Illoldi-Rangel et al. [17] determined that the ecological suitability determined by climatic and environmental variables of ticks related to the transmission of Lyme disease of the genus *Ixodes* and *Amblyomma cajennense* can be used to assess the risk of vector-borne diseases even in poorly studied sites such as Mexico. Feria-Arrollo et al. [18] describe how the distribution of *Ixodes scapularis* responds positively to climate change, expanding its geographical distribution and therefore increasing the risk of contact between this important vector with humans and livestock. 

Many vector-borne pathogens such as Lyme disease, malaria, Chagas disease, or Leishmaniosis simultaneously use several arthropod species to reach their ultimate hosts [19,20,21,22]. The assessment of ecological niche models at a genus level can be a useful tool to identify areas of importance for the dynamics of vector-borne diseases, for the identification of areas of higher risk of contact, or for the delimitation of sanitary control fences to prevent the emergence of a potential infectious outbreak. Strategies intended to contain outbreak episodes are usually expensive and difficult to implement [23], and modeling techniques can be very useful in the design of more efficient sentinel strategies. 

In this study, we estimated the potential distribution of four genera of ticks of medical and veterinary importance in Mexico using nine modeling algorithms in order to identify areas of greater suitability for their presence.

## 2. Materials and Methods 

### 2.1. Tick Occurrence Data

For this study, an exhaustive search was conducted of the presence data of the genera *Amblyomma*, *Ixodes*, *Dermacentor*, and *Rhipicephalus* in Mexico. This search was complicated by the controversial systematics and the difficult taxonomic diagnosis of the group as well as the general lack of study of the tick species of Mexico, with many species having a single record for the entire country. To reduce problems associated with misidentification, we cross-checked all records with the publications of the national collection of mites of the National Autonomous University of Mexico (CNAC-UNAM) [24,25,26]. It is important to note that many tick records in Mexico do not possess appropriate georeferencing, ambiguously indicating their location at the municipal or state level, data useless for the generation of species-specific ecological niche models. The last criterion that we used to refine the database for each genus of tick was its occurrence on a native host. This assumption eliminated many records that are associated with the movement and concentration of livestock in central feedlots collection centers, which could strongly over-fit the predicted distribution in the resulting models.

Each of the four data sets was calibrated and validated according to the training ratio proposed by Huberty [27], which consists of using 70% of the randomly selected records as the training set and the remaining 30% as the evaluation set of each model.

### 2.2. Pseudoabsence Data

Since real absences are difficult to obtain and various ENM need absences, we calculated random pseudoabsence records which is considered a robust method for the algorithms used [28]. We utilized Ecospat Package in R [29] with the following criteria: the proportion of one presence to 10 pseudoabsences to avoid extremely unbalanced samples of presence-absence [30], excluding points of presence, with a minimum distance of 1 km between them and the presence points, and within the calibration area [31].

### 2.3. Environmental Variables 

We calibrated the ecological niche models of the four tick genera in Mexico using two sets of bioclimatic variables. The bioclimatic variables were downloaded from WorldClim (www.worldclim.org) at ~1 km^2^ spatial resolution [32]. Both climatic sets were chosen based on tick biology [33], and of these, highly correlated variables were removed from the analysis following a Pearson correlation test [34]. In addition to the climatic information, we also included two categorical variables that have been proposed as important environmental factors related to tick survival: the type of soil (www.inegi.org.mx/temas/edafologia/) and land use and vegetation (www.inegi.org.mx/temas/usosuelo/). We resampled both of these categorical layers to match the spatial resolution of the WoldClim layers. 

The mean diurnal range, the annual precipitation, the precipitation of driest month, the precipitation seasonality, the type of soil, and the type of land use and vegetation were used to generate the ecological niche models for the four tick genera. To complement the aforementioned additional variables for the *Amblyomma* genus included the minimum temperature of the coldest month and the precipitation of the warmest quarter. For the three remaining genera, we added the maximum temperature of the warmest month, the mean temperature of the wettest quarter, and the precipitation of the wettest quarter (Table 1). The extent of the model calibration area had a strong effect on ENM results [35]. We selected the same polygon for all genera considering Mexico’s political borders. Although the four genera have a larger distribution than our surveyed area, we considered that expanding the models outside the area of interest would result in the loss of the fine-scale aspects of the analysis for Mexico.

### 2.4. Ecological Niche Modeling

The ecological niche models for the four tick genera were estimated with nine different algorithms using seven models included in SDM package in R software v. 3.5 [36], maximum entropy (MAXENT) with ENMEval package [37], and generalized additive model (GAM) with the GAM package [38]. GAM was calibrated out of the sdm package because the number of species-specific tick records considered was too small to predict successfully their distribution. The modeling algorithms developed were Bioclimatic envelope (BIOCLIM) [20], maximum entropy (MAXENT) [39,40], generalized linear models (GLM) [41,42], multivariate adaptive regression splines (MARS) [43], classification and regression trees (CART) [44], mixture discriminant analysis (MDA) [45], random forest (RF) [46], boosted regression trees (BRT) [47,48], and generalized additive model (GAM) [38]. 

BIOCLIM is a simple algorithm that describes the n-dimensional niche of a species in terms of a quadrangular climatic envelope defined by the range of occurrence values at each variable [49,50]. MAXENT is an algorithm that uses the maximum entropy principle and a Bayesian procedure to produce a probability surface where entropy is maximized to reflect the environmental suitability of the geographic area for the species [51]. GLM is an extension of linear models without force data into unnatural scales and related the mean of the response variable and their linear combination of the explanatory variables. GAM is a semi-parametric extension of GLM and the relationship between the mean of the response variable and a “smoothed” function of the explanatory variable(s) [52]. BRT is an ensemble method and combines regression trees and boosting [48]. RF is also a combination of tree predictors and is a classifier. MARS combines regression trees and spline fitting and describes nonlinear relationships between species and environmental variables [53].

We executed Bioclim in the DISMO package [54] and GLM, GAM, BRT, RF, and MARS in SDM package [36]. We ran MAXENT in the ENEval package using default parameters to select feature types and regulation multiplier based on changes in Akaike´s Information Criterion (AIC) [37]. All models were developed in environment R v. 3.5 [55].

The accuracy of each predicted model was evaluated with the area under the curve index (AUC) [56]. Despite the enormous discussion about its relevance as a discriminatory method in the species distribution modeling community, it remains as one of the most used criteria for their evaluation [57,58] because this metric has the ability to discriminate presence from absence (or background) and provides a single measure of performance. The AUC criterion ranges from 0.5 to 1. Values that vary between 0.9 and 1 imply that models have remarkable predictive accuracy, while values that vary between 0.8 and 0.9 have good accuracy and between 0.7 and 0.8 suggest that the models have regular performance [59]. AUC values below 0.7 indicate models of poor and/or failed predictive capacity and should be unconsidered. For all algorithms, we transformed each resulting model into a binary (presence-absence) map by selecting the threshold value where 10% of the occurrence records were left out. We decided to use this threshold criterion to avoid overprediction due to potentially erroneous occurrences [60].

Finally, we added the nine models generated for each genus of ticks in QGIS v. 3.6 to generate consensus maps that show the most suitable sites for these genera of ticks in Mexico.

## 3. Results

**Figure 1:**
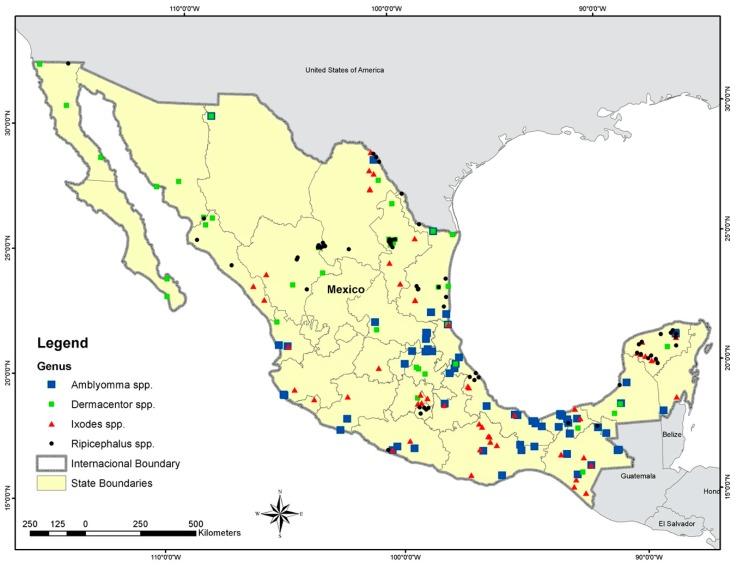
Records used to estimate the ecological niche models for the four tick genera: Black circles indicate the presence of *Rhipicephalus* spp., red triangles indicate the presence of *Ixodes* spp., and green and blue boxes show the presence of *Dermacentor* spp. and *Amblyomma* spp., respectively.

We found 85 total records for *Amblyomma* spp., 44 for *Dermacentor* spp., 71 for *Ixodes* spp., and 72 for *Rhipicephalus* spp. (Figure 1) (Table S1). We selected only the ticks recorded from native hosts because our main concern was the natural distribution of the four genera of ticks in Mexico. This criterion reduced the final number of occurrences because of numerous reports on livestock of uncertain geographic origin. The accuracy for ENM estimated that the models for the tick genera varied from very good (AUC values ≥ 0.8) to regular (AUC values between 0.7 and 0.8) for the nine algorithms used (Table 2). We removed Bioclim from the final model because of its low accuracy for all genera. 

**Figure 2:**
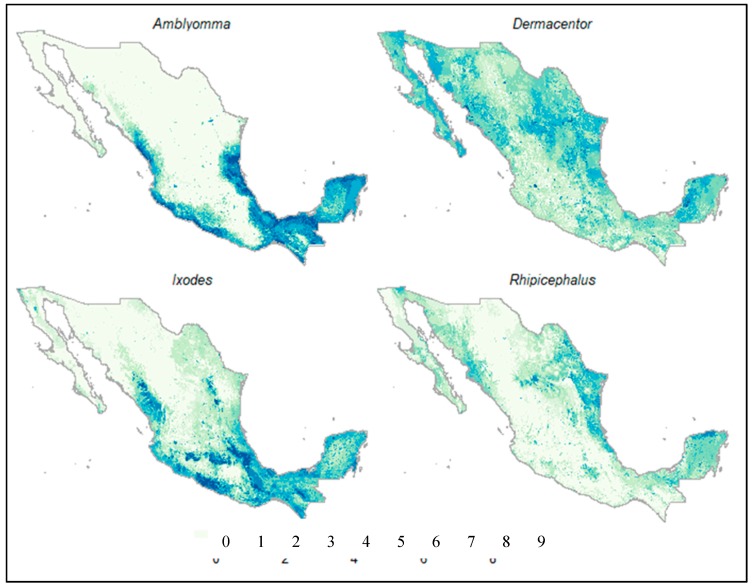
Assembly that brings together the estimated potential distribution of the four tick genera considering the nine algorithms: The darkest areas indicate agreement of the prediction of habitat suitability for each tick genus. The values indicate the number of methods that coincided in determining a certain site as suitable for each genus of tick.

The models of distribution estimated for the genera *Amblyomma* and *Rhipicephalus* had the highest AUC values (Table 2). Overall, the algorithms used to estimate the potential distribution of *Amblyomma* spp. suggested that the areas with greater suitability in Mexico corresponded with the neotropical region of the country (Figure 2). The most important environmental variables for the genus *Amblyomma* were, in order of importance, land use and vegetation, minimum temperature of the coldest month, and precipitation of the warmest quarter (Table S1), which together accounted for about 85% of the registered presences. In contrast, the distribution models for *Rhipicephalus* spp. suggested that the areas of greatest suitability are found in the northeastern portion of the country, in the Yucatan peninsula, and in some areas around the sea of Cortez (Figure 2). The environmental variables that were strongly associated with the presence of the records of *Rhipicephalus* spp. were land use and vegetation, and annual precipitation (Table S2).

The models generated for the *Ixodes* and *Dermacentor* genera had a lower predictive precision. The areas of greatest suitability for the *Ixodes* genus in Mexico are mainly associated with forested areas in the central and southern mountain ranges (Neovolcanic axis and the Sierra Madre Oriental and Sur) and the coastal areas of the Yucatan peninsula (Figure 2). The environmental variables that were associated with the presence of almost 90% of the records are land use, annual rainfall, and average daytime range (Table S2). Finally, the areas suitable for the genus *Dermacentor* in Mexico are more widely distributed than the other three genera (Figure 2). However, the areas of greatest suitability for this genus were mainly located in the Nearctic portion of the country.

## 4. Discussion

The ecological niche models indicated that the four tick genera have different distribution patterns in Mexico (Figure 2). The genus *Amblyomma* in Mexico is represented by 26 species, seven having a Neotropical distribution (*A. nodosum*, *A. oblongoguttatum*, *A. longirostre*, *A. pacar*, *A. pecarium*, *A. rotundatum*, and *A. sabanerae*) and three with a Nearctic distribution (*A. americanum*, *A. coelebs*, and *A.* (=*Robertsicus*) *elaphense*) [61]. Eleven have presence in both biogeographic realms (*A. auricularium*, *A. cajennense*, *A. calcaratum*, *A. dissimile*, *A. imitator*, *A. inornatum*, *A. maculatum*, *A. ovale*, *A. parvum*, *A. triste*, and *A. scutatum*). Five species (*A. humerale*, *A. multipuntum*, *A. tigrinum*, *A. tuberculatum*, and *A. varium*) were not considered in this study because no reliable information was found about their specific location [25]. 

*Amblyomma cajennense* has the most extensive distribution in the country [25], and according to the literature, this species is also one of the most widely distributed in the Americas, ranging from the southern United States to northern Argentina [62]. Ticks from the genus *Amblyomma* are aggressive and are generalist species that participate as vectors of pathogens of medical and veterinary importance. Their presence in the northern hemisphere is associated with warm and humid localities that range in elevation up to 1000 m above sea level and with high ecosystem productivity (NDVI values < 0.56) [63,64,65]. The genus *Amblyomma* has been recorded in 30 of the 32 states of Mexico [25]. The states with the highest number of records are Chiapas, Tamaulipas, and Veracruz, followed by Tabasco, Yucatan, Sinaloa, Nayarit, Colima, and Oaxaca [25,62]. The ecological characteristics described above were consistent with our model, including the prediction being favorable, and the areas associated with the states bordering the Gulf of Mexico and the Pacific Ocean within the Neotropical biogeographical region were predicted as favorable (Figure 2). 

The geographical distribution of *Amblyomma* tends to expand in response to climate change, shifts in land-use, the movement of humans and domestic animals, and the introduction of alien species [66]. Importantly, individuals that have been collected in localities that range between the 1000 and 1500 m above sea levels or in semi-dessert habitats are not documented as reproductive populations [62]. These group of species have a low resistance to desiccation [67] and poor tolerance to temperature variations [62,68,69,70].

The genus *Ixodes* is the most diverse of the family Ixodidae [71]. In Mexico, there are 26 species of the 243 recognized worldwide. However, most of these species are poorly represented in Mexico, represented by only one record within the country, and many of the immature stages of these species remain unknown [24]. *Ixodes ricinus* is recognized as a complex of several species of ticks that together have an almost cosmopolitan distribution (*I. ricinus, I. scapularis, I. pacificus, I. affinis, I. pavloski, I. persulcatus, I. nipponensis, I. gibbosus, I. jellisoni, I. pararicinus,* and *I. nuttallianus*) [72]. Subsequent phylogenetic studies determined that *I. muris, I. minor*, and *I. granulatus* should also be included in the *I. ricinus* complex [73,74]. The most widely distributed species from the complex in Europe are *I. ricinus* and *I. persulcatus*, while in North America, they are *I. scapularis* and *I. pacificus*. These species occupy similar niches in their respective distribution areas [75].

In North America, many of the immature stages of *Ixodes* species can parasitize a large number of hosts that include reptiles, birds, and mammals, whereas *Ixodes* adults tend to be restricted to large mammals (e.g., cervids and carnivores) [76,77,78]. Most *Ixodes* ticks perform vertical movements on the vegetation to reach their host. However, within the genus, it has also been observed that some species search for their hosts in relatively open environments. Regardless of their behavior, our results are consistent with the associations found previously of these ticks to ecosystems with a relatively high percentage of vegetation covering the ground since they have shown to be sensitive to extreme temperatures and dry conditions (Figure 2). For example, it was determined that unfed nymphs reached a 50% mortality at an exposure of −11.6 °C during eight consecutive hours under laboratory conditions [79]. Vegetation was found to generate microenvironmental conditions that prevent the temperature from falling below 0 °C, even during the intensely cold winter periods of the forested environments where these ticks are regularly found. Furthermore, it has been shown that *Ixodes* members are particularly sensitive to high temperatures (~30 °C) and water loss [80], with higher temperatures associated with increased mortality, reduced oviposition success, and host-seeking activity. 

Globally, 36 species of *Dermacentor* are recognized [81], 11 of which are documented in Mexico. In general, *Dermacentor* ticks are found in all biogeographic regions [82], but they are better represented in the Holarctic region. However, *D. albipictus, D. dissimilis, D. halli, D. nitens*, and *D. variabilis* occur in both the Nearctic and Neotropical realms, and *D. imitans* is a Neotropical species. Native artiodactyl and perissodactyl mammals are common hosts of this species. However, *D. albipictus* and *D. variabilis*, which are the most common species in Mexico, are also associated with native carnivora and lagomorpha that inhabit many states of the country [26,83].

As with other tick genera, the distribution of *Dermacentor* is the result of a complex interaction between climate variables, and host and habitat availability [83,84]. Nonetheless, climate constraints seem not to be a limiting factor for *Dermacentor* spp. Dispersal because they have an efficient water balance, which enables them to colonize new environments [80,85]. This resistance to desiccation combined with the association of adult *Dermacentor* ticks with wide distributed hosts [26] could explain the board distribution in Mexico (Figure 2).

*Rhipicephalus* is present in all biogeographic realms. However, there are no endemic species of this genus in the Nearctic and the Neotropical regions [82], which suggests that this genus was probably introduced to the Americas with livestock or other animals from Eurasia and Africa. The genus *Rhipicephalus* contains what are probably the most generalist species, parasitizing amphibians, reptiles, birds, and mammals. Besides high diversity of hosts, it has been suggested that *Rhipicephalus* genus in the Americas also has species that have adapted well to both tropical and temperate ecosystems [86]. Within Mexico, there is a greater proportion of species adapted to tropical ecosystems, with the more suitable areas for this genus in costal ecosystems (Figure 2).

Overall, ticks have physiological requirements which require certain environmental characteristics that are optimal for their development and survival [87]. The localities where these characteristics converge can potentially be part of their geographical distribution [88], and due to their ability to maintain and transmit pathogens of veterinary and medical importance, these localities can be considered as important risk areas [89]. Although potential distribution models are extensively used, it is important to consider that different algorithm can estimate different outcomes [90]. However, the generation of an assembly of the predicted models can help identify areas of agreed suitability for ticks and for the establishment of surveillance and control programs.

Management recommendations should consider that the main limitation of ecological niche models when used to estimate the potential distribution of tick species is the taxonomic inconsistencies widely discussed by different authors [91,92,93]. The geographic distribution of a species is determined by a set of complex ecological, geological, and evolutionary processes of each taxon, so the correct geographic location of a record to a species is crucial for the generation of successful models [18,88,94,95]. We strongly believe that the estimation of genus-based ecological niche models can be useful in approaching the identification of suitable areas for these vectors of medical and veterinary importance.

The knowledge about tick diversity and the ecological features that determine their distribution in Mexico is far from completion. We are currently at a decisive moment for humanity, in which we must not only be able to deal the consequences of the changes we have exerted directly on biodiversity, land use, and climate but also must understand how these modifications influence complex processes such as vector dynamics and the diseases they transmit. The technical improvements to ecological niche modeling and the generation of quality databases may assist the decision-making processes during this period of uncertainty to prevent the emergence of infectious outbreaks that threaten human wellbeing and animal health.

## Figures and Tables

**Table 1:** Climatic and environmental variables used for the generation of ecological niche models of the four tick genera.

Genera	*Amblyomma*	*Dermacentor*, *Ixodes* and *Rhipicephalus*
Annual mean temperature		•
Mean diurnal range	•	•
Temperature Seasonality		•
Max temperature of warmest month		•
Min temperature of coldest month	•	
Mean temperature of wettest quarter		•
Annual precipitation	•	•
Precipitation of driest month	•	•
Precipitation seasonality	•	•
Precipitation of wettest quarter		•
Precipitation of the warmest quarter	•	
Type of soil	•	•
Type of land use and vegetation	•	•

**Table 2:** Area under the curve index (AUC) values for each of the models generated by tick genus.

Algorithm	*Amblyomma* spp.	*Dermacentor* spp.	*Ixodes* spp.	*Rhipicephalus* spp.
BIOCLIM	0.706	0.664	0.669	0.772
BRT	0.905	0.872	0.892	0.921
CART	0.913	0.856	0.883	0.965
MDA	0.883	0.797	0.789	0.869
GAM	0.93	0.871	0.962	0.98
GLM	0.888	0.804	0.792	0.878
MARS	0.92	0.941	0.947	0.962
MAXENT	0.901	0.840	0.918	0.931
RF	0.999	0.996	0.994	0.999

BRT: Boosted Regression Trees, CART: Classification And Regression Tree, MDA: Mixture Discriminant Analysis, GAM: Generalized Additive Models, GLM: Generalized Linear Models; MARS: Multivariate adaptive regression spline, Maxent: Maximum entropy, RF: Random Forest

## Supplementary Materials

The following are available online at https://www.mdpi.com/2076-2615/10/4/649/s1, Table S1: Records used for the generation of ecological niche models for the four genera of ticks. Table S2: Climatic and environmental variables analyzed to make ecological niche models for the four tick genera.
