# Supplementary material for: Ecological Niche Models of Four Hard Tick Genera (Ixodidae) in Mexico
Source: Animals (Basel). 2020 Apr 9;10(4):649. doi: 10.3390/ani10040649 (PMC7222792; doi:10.3390/ani10040649)
Supplement: Supplementary file 1 [file animals-10-00649-s001.pdf]

animals

Supplementary Files: Ecological Niche Models of Four Hard Tick Genera (Ixodidae) in Mexico

Emilio Clarke-Crespo [1,2], Claudia N. Moreno-Arzate [3] and Carlos A. López-González [1,*]

Table S1. Records used for the generation of ecological niche models for the four genera of ticks.

Tick Genus	Longitude	Latitude
Amblyomma	-90.716667	18.616667
Amblyomma	-90.416667	19.416667
Amblyomma	-95.866667	16.033333
Amblyomma	-92.716667	15.916667
Amblyomma	-93.116667	16.752778
Amblyomma	-91.008917	16.755833
Amblyomma	-100.43333	17.2
Amblyomma	-99.509483	17.250187
Amblyomma	-105.04436	19.49807
Amblyomma	-105.04411	19.497916
Amblyomma	-105.044	19.498124
Amblyomma	-105.04228	19.497718
Amblyomma	-105.04222	19.496922
Amblyomma	-105.04026	19.496349
Amblyomma	-105.04272	19.497793
Amblyomma	-105.0424	19.499057
Amblyomma	-105.04376	19.499643
Amblyomma	-105.07212	19.527222
Amblyomma	-102.64188	18.063084
Amblyomma	-105.28556	21.539722
Amblyomma	-95.051111	17.226389
Amblyomma	-94.453971	17.122241
Amblyomma	-98.160795	19.022078
Amblyomma	-97.678611	20.438056
Amblyomma	-99.819684	20.697599
Amblyomma	-88.982778	18.1925
Amblyomma	-101.05191	22.437763
Amblyomma	-98.836389	21.935278
Amblyomma	-91.426667	17.4725
Amblyomma	-91.75375	17.733322
Amblyomma	-92.946293	17.567107
Amblyomma	-92.937789	17.565807
Amblyomma	-92.938857	17.562188
Amblyomma	-92.597487	18.133358
Amblyomma	-93.016578	18.133333
Amblyomma	-93.383305	17.84998
Amblyomma	-93.210833	18.283611
Amblyomma	-93.296111	18.333889
Amblyomma	-98.591944	22.754167
Amblyomma	-97.926111	22.660278
Amblyomma	-94.477755	18.145282
Amblyomma	-94.405	18.149722
Amblyomma	-94.350729	18.045111
Amblyomma	-94.092778	17.91
Amblyomma	-95.202397	18.45

Amblyomma	-95.200797	18.447797
Amblyomma	-95.203321	18.446198
Amblyomma	-96.380537	18.852444
Amblyomma	-95.092083	18.414544
Amblyomma	-97.46043	20.875732
Amblyomma	-88.158333	21.1425
Amblyomma	-88.164548	21.148285
Amblyomma	-88.167759	21.190772
Amblyomma	-98.606389	21.171944
Amblyomma	-98.9	21.166667
Amblyomma	-99.477487	21.210078
Amblyomma	-98.860278	21.672778
Amblyomma	-98.8	21.916667
Amblyomma	-98.8	21.916667
Amblyomma	-98.795833	21.265556
Amblyomma	-98.583395	22.756758
Amblyomma	-97.880178	22.228424
Amblyomma	-97.880178	22.228424
Amblyomma	-92.974765	17.989337
Amblyomma	-92.133333	16.25
Amblyomma	-93.211161	18.284402
Amblyomma	-93.284765	18.36518
Amblyomma	-98.324827	26.045394
Amblyomma	-105.04427	19.498281
Amblyomma	-102.35	18.51667
Amblyomma	-104.9	21.5
Amblyomma	-92.95333	17.548889
Amblyomma	-91.008917	16.756083
Amblyomma	-96.609444	17.048056
Amblyomma	-90.949033	16.781
Amblyomma	-100.22774	17.342042
Amblyomma	-105.04389	19.495739
Amblyomma	-97.894722	20.254167
Amblyomma	-97.616667	20.616667
Amblyomma	-97.615932	20.616223
Amblyomma	-95.202989	18.446198
Amblyomma	-94.999929	16.99967
Amblyomma	-100.9	29.05
Amblyomma	-92.974765	17.989337
Amblyomma	-108.5025	30.863889
Dermacentor	-97.61547	20.615847
Dermacentor	-91.017748	18.237601
Dermacentor	-90.748461	18.595424
Dermacentor	-105.37683	22.494014
Dermacentor	-108.61488	26.412188
Dermacentor	-92.589135	17.759399
Dermacentor	-97.880176	22.226199
Dermacentor	-116.79584	32.474666
Dermacentor	-115.34712	30.925793
Dermacentor	-108.31743	26.709744
Dermacentor	-113.55934	28.950236
Dermacentor	-110.26631	24.160111
Dermacentor	-110.23134	23.446531
Dermacentor	-103.33454	24.489415
Dermacentor	-99.303415	19.30446
Dermacentor	-103.51277	25.524448

Dermacentor	-104.67563	24.003259
Dermacentor	-99.22157	20.479278
Dermacentor	-98.949592	20.260624
Dermacentor	-99.315109	20.544049
Dermacentor	-100.99691	22.127954
Dermacentor	-109.9086	28.154579
Dermacentor	-110.91381	27.901171
Dermacentor	-115.34712	30.925793
Dermacentor	-92.499379	15.999834
Dermacentor	-100.26228	25.674199
Dermacentor	-100.05003	25.78797
Dermacentor	-100.13378	25.654289
Dermacentor	-100.15355	25.65597
Dermacentor	-100.26593	25.564913
Dermacentor	-100.25199	25.682547
Dermacentor	-100.32654	25.799027
Dermacentor	-100.28205	25.750983
Dermacentor	-108.71653	26.724111
Dermacentor	-97.775826	23.786171
Dermacentor	-98.206334	23.767346
Dermacentor	-88.568826	20.684065
Dermacentor	-100.72339	28.222243
Dermacentor	-100.13312	27.240027
Dermacentor	-98.32744	26.053668
Dermacentor	-97.463118	25.875699
Dermacentor	-108.5025	30.863889
Dermacentor	-108.5025	30.863889
Dermacentor	-108.5025	30.863889
Ixodes	-93.366667	16.75
Ixodes	-92.898333	15.427778
Ixodes	-88.176944	21.018889
Ixodes	-89.716667	20.48333
Ixodes	-88.164722	21.1425
Ixodes	-88.164722	21.1425
Ixodes	-89.288056	20.201944
Ixodes	-89.533889	20.395278
Ixodes	-89.616667	20.966667
Ixodes	-89.856944	20.549722
Ixodes	-89.108333	20.235278
Ixodes	-92.422889	16.578694
Ixodes	-92.422889	16.578694
Ixodes	-101.12	27.84833
Ixodes	-96.028889	17.256944
Ixodes	-96.368639	17.628639
Ixodes	-96.368639	17.628639
Ixodes	-96.733333	18.15
Ixodes	-92.466667	18.133333
Ixodes	-92.134167	16.251111
Ixodes	-92.794167	15.715278
Ixodes	-100.43306	17.20667
Ixodes	-100.43306	17.20667
Ixodes	-96.294722	17.400833
Ixodes	-96.616223	18.017688
Ixodes	-88.395278	18.676944
Ixodes	-96.315278	17.609444
Ixodes	-97.146389	19.690833

Ixodes	-95.214167	18.441389
Ixodes	-99.183331	19.416669
Ixodes	-101.02972	29.363056
Ixodes	-101.1214	28.634444
Ixodes	-99.905556	23.969722
Ixodes	-99.905556	23.969722
Ixodes	-97.103611	19.638333
Ixodes	-98.9	19.266667
Ixodes	-100.34194	24.826389
Ixodes	-98.1833	18.9667
Ixodes	-96.745637	17.107997
Ixodes	-96.368639	17.628639
Ixodes	-99.686924	17.556351
Ixodes	-97.146389	19.690833
Ixodes	-92.666064	18.525371
Ixodes	-92.424114	15.148906
Ixodes	-92.666064	18.525371
Ixodes	-103.75507	19.319752
Ixodes	-97.145729	19.688371
Ixodes	-99.68625	17.555246
Ixodes	-104.60229	19.720846
Ixodes	-97.15	16.1
Ixodes	-97.860637	22.233012
Ixodes	-100.92025	28.490424
Ixodes	-104.87172	21.493119
Ixodes	-106.42858	23.943771
Ixodes	-105.85243	24.437536
Ixodes	-105.93638	23.396831
Ixodes	-99.15001	19.085519
Ixodes	-99.686924	17.556351
Ixodes	-99.686924	17.556351
Ixodes	-99.686924	17.556351
Ixodes	-99.312957	19.055335
Ixodes	-97.146389	19.690833
Ixodes	-99.275996	23.275954
Ixodes	-102.3181	19.416943
Ixodes	-97.145729	19.688371
Ixodes	-99.686924	17.556351
Ixodes	-100.95314	20.560355
Ixodes	-99.177376	25.788189
Ixodes	-101.12056	27.871223
Ixodes	-99.272444	23.279861
Ixodes	-99.310254	19.058455
Rhipicephalus	-104.44861	25.130408
Rhipicephalus	-104.04405	23.826372
Rhipicephalus	-104.48771	25.044769
Rhipicephalus	-103.49988	25.623897
Rhipicephalus	-103.53416	25.531589
Rhipicephalus	-89.622584	20.972245
Rhipicephalus	-88.201909	20.690602
Rhipicephalus	-89.711008	20.486978
Rhipicephalus	-88.15145	21.143315
Rhipicephalus	-88.178914	21.021234
Rhipicephalus	-88.793746	21.200455
Rhipicephalus	-108.99092	25.794271
Rhipicephalus	-107.40462	24.794922

Rhipicephalus	-103.26712	25.640578
Rhipicephalus	-103.21386	25.580319
Rhipicephalus	-103.25981	25.582989
Rhipicephalus	-103.23081	25.586639
Rhipicephalus	-103.27538	25.572169
Rhipicephalus	-103.30592	25.721947
Rhipicephalus	-102.15044	25.440189
Rhipicephalus	-103.47078	25.583789
Rhipicephalus	-89.415797	20.304478
Rhipicephalus	-88.270556	21.296389
Rhipicephalus	-89.85871	20.555246
Rhipicephalus	-89.107427	20.234472
Rhipicephalus	-99.235525	18.919435
Rhipicephalus	-103.40253	25.526413
Rhipicephalus	-103.5328	25.540184
Rhipicephalus	-103.48661	25.588123
Rhipicephalus	-89.283	20.4001
Rhipicephalus	-103.49726	25.584738
Rhipicephalus	-89.749711	20.886004
Rhipicephalus	-100.31592	25.681652
Rhipicephalus	-100.252	25.590804
Rhipicephalus	-100.25577	25.674508
Rhipicephalus	-100.28552	25.74628
Rhipicephalus	-100.18659	25.769446
Rhipicephalus	-100.31307	25.791262
Rhipicephalus	-100.335	25.701108
Rhipicephalus	-100.17571	25.472109
Rhipicephalus	-100.05002	25.787505
Rhipicephalus	-115.45235	32.62039
Rhipicephalus	-108.70071	26.681734
Rhipicephalus	-89.050688	20.070575
Rhipicephalus	-96.850501	19.93331
Rhipicephalus	-97.037361	20.066574
Rhipicephalus	-96.776938	20.206928
Rhipicephalus	-96.655103	20.028981
Rhipicephalus	-99.177231	23.847147
Rhipicephalus	-99.105496	23.731608
Rhipicephalus	-99.120819	23.712416
Rhipicephalus	-97.880161	24.092748
Rhipicephalus	-97.897463	23.349695
Rhipicephalus	-100.62738	17.213077
Rhipicephalus	-88.275089	21.300932
Rhipicephalus	-88.138099	21.15298
Rhipicephalus	-88.367637	21.21067
Rhipicephalus	-90.712762	19.344398
Rhipicephalus	-98.86376	18.876652
Rhipicephalus	-98.964196	18.821545
Rhipicephalus	-99.205244	18.660383
Rhipicephalus	-99.043563	18.891682
Rhipicephalus	-98.011358	22.965006
Rhipicephalus	-100.77052	29.165522
Rhipicephalus	-100.64869	28.974799
Rhipicephalus	-98.942709	26.361062
Rhipicephalus	-100.89957	29.306208
Rhipicephalus	-99.665651	27.637634
Rhipicephalus	-98.20352	23.765245

Rhipicephalus	-91.77643	17.795835
Rhipicephalus	-92.974765	17.989337
Rhipicephalus	-92.974765	17.989337

Table S2. Climatic and environmental variables analyzed to make ecological niche models for the four tick genera.

Variable	Unit	Resolution	Type	Reference
Annual mean temperature	°C	30 s (~1 km^2)	Continuous	www.worldclim.org
Mean diurnal range	°C	30 s (~1 km^2)	Continuous	www.worldclim.org
Isothermality	Dimensionless	30 s (~1 km^2)	Continuous	www.worldclim.org
Temperature seasonality	°C	30 s (~1 km^2)	Continuous	www.worldclim.org
Maximum temperature of warmest month	°C	30 s (~1 km^2)	Continuous	www.worldclim.org
Minimum temperature of coldest month	°C	30 s (~1 km^2)	Continuous	www.worldclim.org
Temperature annual range	°C	30 s (~1 km^2)	Continuous	www.worldclim.org
Mean temperature of wettest quarter	°C	30 s (~1 km^2)	Continuous	www.worldclim.org
Mean temperature of driest quarter	°C	30 s (~1 km^2)	Continuous	www.worldclim.org
Mean temperature of warmest quarter	°C	30 s (~1 km^2)	Continuous	www.worldclim.org
Mean temperature of coldest quarter	°C	30 s (~1 km^2)	Continuous	www.worldclim.org
Annual precipitation	mm	30 s (~1 km^2)	Continuous	www.worldclim.org
Precipitation of wettest month	mm	30 s (~1 km^2)	Continuous	www.worldclim.org
Precipitation of driest month	mm	30 s (~1 km^2)	Continuous	www.worldclim.org
Precipitation seasonality	Dimensionless	30 s (~1 km^2)	Continuous	www.worldclim.org
Precipitation of wettest quarter	mm	30 s (~1 km^2)	Continuous	www.worldclim.org
Precipitation of driest quarter	mm	30 s (~1 km^2)	Continuous	www.worldclim.org
Precipitation of warmer quarter	mm	30 s (~1 km^2)	Continuous	www.worldclim.org
Precipitation of coolest quarter	mm	30 s (~1 km^2)	Continuous	www.worldclim.org
Type of soil	area	1:250 000	Categorical	www.inegi.org.mx/temas/edafologia/
Type of land use and vegetation	area	1:250 000	Categorical	www.inegi.org.mx/temas/usodesuelo/
